# Monitoring breastfeeding indicators in high‐income countries: Levels, trends and challenges

**DOI:** 10.1111/mcn.13137

**Published:** 2021-01-06

**Authors:** Juliana S. Vaz, Maria Fatima S. Maia, Paulo A. R. Neves, Thiago M. Santos, Luís Paulo Vidaletti, Cesar Victora

**Affiliations:** ^1^ International Center for Equity in Health Universidade Federal de Pelotas Pelotas Brazil; ^2^ Faculty of Nutrition Universidade Federal de Pelotas Pelotas Brazil; ^3^ Universidade Federal do Rio Grande ‐ FURG Rio Grande Brazil

**Keywords:** breastfeeding, developed countries, global health, health policy, socio‐economic factors, survey methods

## Abstract

Monitoring indicators of breastfeeding practices is important to protect and evaluate the progress of breastfeeding promotion efforts. However, high‐income countries lack standardized methodology to monitor their indicators. We aimed to update and summarize nationally representative annual estimates of breastfeeding indicators in high‐income countries and to describe methodological issues pertaining to the data sources used. A review was conducted through population‐based surveys with nationally representative samples or health reports from nationally representative administrative data of electronic surveys or medical records. Methodological aspects and rates of all breastfeeding indicators available were summarized by country. The median and annual growth of breastfeeding in percentage points within countries with time‐series data were estimated. Data from 51 out of 82 high‐income countries were identified. The data were obtained through surveys (*n* = 32) or administrative data (*n* = 19). Seventy‐one percent of countries have updated their indicators since 2015. Ever breastfed was the indicator most frequently reported (*n* = 46), with a median of 91%. By 6 months of age, the median equals 18% for exclusive and 45% for any breastfeeding. At 12 months, the median of continued breastfeeding decreased to 29%. The annual growth rate for ever breastfed, exclusive and any breastfeeding at 6 months and continued at 12 months varied from 1.5 to −2.0, 3.5 to −3.1, 5.0 to −1.0 and 5.0 to −1.9, respectively, with positive changes for most countries. Stronger interventions are needed to promote breastfeeding in high‐income countries as a whole, and investments are required to monitor trends with standardized methodologies.

**Key messages**

- Current breastfeeding practices in most high‐income countries fall well short of international recommendations of exclusive breastfeeding under 6 months and continued breastfeeding until 2 years.
- Thirty‐six out of 51 high‐income countries have updated their indicators since 2015, and eight have collected breastfeeding indicators for more than two decades.
- National censuses of children estimated from maternity hospitals or follow‐up in primary care services have replaced national health and child surveys in high‐income countries.
- Lack of standardized methodologies and definitions affect the comparability of breastfeeding indicators of high‐income countries, especially related to the variability of time frame to characterize exclusive breastfeeding under 6 months.

## 1 INTRODUCTION

Monitoring indicators of breastfeeding practices is important to protect and evaluate the progress of breastfeeding promotion efforts. Despite the prevalence of breastfeeding initiation being over 80% in most high‐income countries, a drastic drop in breastfeeding rates is observed within the first 6 months of life, especially in the case of exclusive breastfeeding (Victora et al., 2016). Longer breastfeeding reduces the infant risk of infectious morbidity and mortality, dental malocclusions and probably the risk of obesity and type 2 diabetes, and it increases the child's intelligence (Victora et al., 2015), an effect that persists until adult life. For mothers, breastfeeding may prevent breast and ovarian cancer and may prevent the risk of diabetes (Victora et al., 2016). The Lancet Breastfeeding Series in 2016 highlighted the many benefits of breastfeeding to mothers and children, both in poor and in rich countries (Victora et al., 2016). Nevertheless, only a small proportion of all children receive any breast milk at 12 months of age (Sarki, Parlesak, & Robertson, 2019; Victora et al., 2016).

Indeed, there has been growing attention around the failure to protect, promote and support breastfeeding in high‐income settings (Bagci Bosi, Eriksen, Sobko, Wijnhoven, & Breda, 2016; Mirkovic, Perrine, Scanlon, & Grummer‐Strawn, 2014). Mothers and children in these countries are placed in a vulnerable position as a result of aggressive marketing by the infant formula industry. The violation of the International Code of Marketing of Breast Milk Substitutes is frequently reported (Baker et al., 2016; Grummer‐Strawn, Holliday, Jungo, & Rollins, 2019; Theurich et al., 2019). Few hospitals adopt baby‐friendly practices aimed at promoting the successful initiation of breastfeeding (Theurich et al., 2019). Additionally, there is wide variability among high‐income countries regarding the implementation of recognized interventions to support breastfeeding after hospital discharge, such as counselling by a nurse, trained lactation counsellor, post‐discharge telephone calls and home visits (Rollins et al., 2016; Skouteris et al., 2014). There are also marked differences in maternal schooling levels and family income in breastfeeding indicators within countries of the World Health Organization (WHO) European Region (Bærug et al., 2017; Hughes, 2015; Sarki et al., 2019) and in North America (Hughes, 2015). Another critical issue is paid parental leave, which also differs widely among high‐income countries. The length and level of maternal and parental leaves are important determinants to support mothers and to prolong breastfeeding rates (Theurich et al., 2019).

However, few high‐income countries monitor their indicators according to the current WHO guidelines (Sarki et al., 2019; Theurich et al., 2019; Victora et al., 2016). The lack of standardized surveys and inconsistencies in data collection among these countries prevent WHO breastfeeding recommendations from being monitored. This may also delay the timely implementation of appropriate policy actions to protect and promote breastfeeding (Sarki et al., 2019; Theurich et al., 2019; Victora et al., 2016). We aimed to update and summarize nationally representative annual estimates of breastfeeding indicators in high‐income countries and to describe methodological issues pertaining to the data sources used, providing a critical appraisal of available data.

## 2 METHODS

Our analyses were based on nationally representative breastfeeding indicators from countries classified as high‐income economies. The potential list of countries was obtained from the World Bank database that classifies global economies based on levels of gross national domestic product per capita (World Bank, 2019). According to the 2018/2019 fiscal year review, 79 countries (including territories) were defined as high‐income economies. For the United Kingdom, we sought to obtain data for each of the four constituent countries (England, Wales, Northern Ireland and Scotland). Thus, our search was focused on 82 countries and territories.

Data sources were restricted to population‐based surveys with nationally representative samples or health reports from nationally representative administrative rates obtained from electronic surveys or medical records (e.g., maternity hospitals and primary health care facilities). We used the search engines PubMed, Google Scholar and other sources, as described in Appendix A. The search was conducted in March 2018 and updated in November 2019. We searched the reference lists in all documents to identify past publications and websites from official institutions with additional information. We also activated e‐mail alerts from main journals in the area of public health and maternal child nutrition.

Additionally, we e‐mailed research institutions, government health departments, national breastfeeding associations and experts in the field from several countries. From these contacts, we requested information about recent and upcoming surveys, as well as on the existence of other possible data sources with national representative samples (e.g., health reports of administrative data).

We selected documents with sufficiently detailed methodological descriptions. We only incorporated breastfeeding rates published in country card reports and websites from international agencies and civil society organizations after accessing the original complete references. The documents were obtained in a diversity of national languages and, if necessary, we used the Google Translate website. In some cases, a brief report and tables in English were obtained when contacting national researchers by e‐mail.

### 2.1 Data extraction

We extracted all available annual data points into an Excel spreadsheet. Countries were characterized according to the type of institution that produced the data, data source type (survey or electronic data), year, follow‐up, target population and response rate (in case of electronic data, percent of health units that submitted data reports).

We carefully reviewed the surveys or health statistical report methodology to identify changes in study design or time frame estimation over time that could affect the retrospective comparison of annual data. For countries with more than one data source, we prioritized those with the highest national representativeness and those provided by governmental institutions.

### 2.2 Breastfeeding indicators

Several national surveys and health reports did not estimate breastfeeding indicators according to the internationally standardized definitions (WHO, 2008, 2018). To allow comparison, we grouped the available indicators into specific breastfeeding variables as follows:

- Early initiation of breastfeeding within the first hour of birth, using the international recommendation.
- Ever breastfed: infants reported having been breastfed, even if for a short period, including breastfeeding at the hospital, or any breastfeeding up to 2 weeks after birth. If needed, we estimated ‘ever breastfed’ by the complement of the percent of children never breastfed. Five countries (Antigua and Barbuda, Aruba, Israel, Japan and Latvia) that did not have data on ever breastfeeding but reported on the percent of children receiving any breast milk at 1, 2 or 3 months; these countries were analysed separately.
- Exclusive breastfeeding was extracted according to the variety of time frames presented in national surveys and reports: 1 week, 2 weeks, 1 month, 2 months, 3 months, 4 weeks, 5 months, and 6 months, 0–3 months and 0–5 months.
- Any breastfeeding at 6 months, including any breastfeeding at 5–6 months.
- Continued breastfeeding at 1 year, including estimations at 9 to 11 months, >10 to 12 months, <12 months, 12 months, 12 to 15 months and 13 to 15 months.
- Continued breastfeeding at 2 years, including estimations at 20 to 23 months, 21 to 23.9 months and 24 months.

A detailed summary of the WHO indicators and breastfeeding rates grouped is presented in Table 1.

**TABLE 1:** Summary of breastfeeding indicators reported by high‐income countries (*n* = 51)

Breastfeeding indicators (WHO)	National definitions	Number of countries
Early Initiation of breastfeeding (% of children born in the last 24 months who were put to the breast within 1 h of birth)	Breastfed within 1 h after birth	9
Ever breastfed (% of children born in the last 24 months who were ever breastfed, even if for a short period)	Ever breastfed	28
	Never breastfed^a^	5
	Breastfeeding at hospital	6
	Breastfed at 1–2 weeks	2
	Breastfed at 1 month	2
	Breastfed up to 2 months	2
	Breastfed up to 3 months	1
Exclusive breastfeeding (% of children 0–5 months who are fed exclusively on breast milk, or ‘during the previous day’. Further disaggregated recommended is 0–1 month, 2–3 months, 0–3 months and 4–5 months)	1 week	6
	2 weeks	4
	1 month	17
	2 months	15
	3 months	20
	0–3 months	2
	4 months	14
	5 months	10
	0–5 months	7
	6 months	30
Continued breastfeeding at 1 year (% of children aged 12–15 months who received any breastmilk during the previous day)	9–11 months	1
	>10–12 months	1
	<12 months	1
	12 months	17
	12–15 months	4
	13–15 months	1
Continued breastfeeding at 2 years (% of children 20–23 months of age who received any breastmilk during the previous day)	20–23 months	2
	21–23.9 months	1
	24 months	2

^a^ ‘Ever breastfed’ was estimated by the complement of the percent of children never breastfed.

### 2.3 Statistical analysis

Descriptive analyses were performed with Stata 16.0 and R software, version 4.0.2. We presented a methodological description of the data extracted (i.e., source of data, annual updates, population surveyed and response rate) and the number of indicators by country and data source. Bar charts were prepared with the last updated breastfeeding rates per indicator. For countries with two estimates for the same indicator over time, we used the Wilcoxon signed‐rank test to assess whether there was a significant change over time in the breastfeeding indicator when all countries with two data points were considered.

Annual growth analyses in percentage points (pp) were carried out by calculating the difference between the last and first annual rates divided by the difference in years between rates. Analyses were restricted to countries with annual rates estimated in a similar time frame and without differences in methodology that restricted comparisons between years. Graphs were displayed for each indicator to allow visual comparisons across countries.

### 2.4 Ethical considerations

The datasets used are publicly available with no identifiable information. Ethical clearance for conducting the surveys were the responsibility of the institutions conducting the survey.

## 3 RESULTS

We obtained data from 51 out of 82 high‐income countries and territories. The time period covered by the data points ranged from 1986 to 2019. National breastfeeding indicators were estimated through breastfeeding surveys (*n* = 4), infant surveys (*n* = 13), infant immunization surveys (*n* = 5), national health surveys (*n* = 10), national health statistical reports summarized from maternity hospitals (*n* = 3) and primary health facilities (*n* = 16). Thus, data were gathered from 32 national surveys and 19 electronic medical record systems (Table 2, Appendix B).

**TABLE 2:** Methodological aspects of breastfeeding data source from high‐income countries (*n* = 51)

Country/territories	First year	Last year	Type of data	Study population	Follow‐up^a^	Response rate (%)^b^
**Region of the Americas**						
Antigua and Barbuda	1995	2006	National Health survey	Children under 5 years	‐	ND
Aruba	2002	2010	National Health survey	ND	‐	ND
Bahamas	2001	2001	National Health survey	Children 0–24 months	‐	ND
Canada	2000	2018	National Health survey	Women 15–55 years who gave birth in the last 5 years	Annual	96
Chile	2005	2016	Electronic data (primary health care)	Children under 6 years	Annual	94
Puerto Rico	2013	2015	Infant immunization survey	Children 19–35 months	Annual	33
United States	2000	2016	Infant immunization survey	Children 19–35 months	Annual	33
Uruguay	1996	2011	Breastfeeding survey	Children 0–24 months	^d^	94
Virgin Islands	2014	2015	Infant immunization survey	Children 19–35 months	Annual	33
**Western Pacific Region**						
Australia	1995	2018	National Health survey	Children 0–3 years	3 years	76
Brunei Darussalam	2009	2009	Infant survey	Children 0–5 years	‐	ND
Guam	2014	2016	Infant immunization survey	Children 19–35 months	Annual	33
Japan	1985	2015	Infant survey	Children up to 2 years	5 years	60
Korea, Republic	2003	2018	National Health survey	Married women (15–45 years) who gave birth in the last 3 years	3 years	ND
New Zealand	1999	2017	Electronic data (Maternity hospitals)	Birth to 6 weeks	Annual	95
Palau	2010	2018	Electronic data (primary health care)	Women who gave birth in the last 4 years	Annual	ND
Singapore	2001	2011	Breastfeeding survey	Women who gave birth and followed up to 6 months	^d^	46
**European Region**						
Austria	2006	2006	Infant survey	Children followed up to 12 months	‐	ND
Belgium	2012	2012	Infant immunization survey	Children 18–24 months	‐	ND
Croatia, Republic	2005	2016	Electronic data (primary health care)	Children 0–12 months	Annual	97/90
Cyprus	2014	2017	Electronic data (Maternity hospitals)	Birth	Annual	99
Czech Republic	2000	2017	Electronic data (primary health care)	Children 0–12 months	Annual	ND
Denmark	2012	2017	Electronic data (primary health care)	Children 0–12 months	Annual	99
England	2003	2018	Electronic data (primary health care)	Birth to 8 weeks	Annual	96
Estonia	1998	2019	Electronic data (primary health care)	Children 0–12 months	Annual	99
Finland	1995	2019	Infant survey	Children 0–12 months	9 years	22
France	2010	2016	Infant survey	Children followed up from birth to 12 months	‐	94
Germany	2009	2012	Infant survey	Children under 6 years	‐	39
Greece	2007	2017	Breastfeeding survey	Children 6–9 months	‐	63
Iceland	1999	2008	Electronic data (primary health care)	Children 0–12 months	Annual	60
Ireland, Republic	2007	2016	Electronic data (primary health care)	Birth to 6 weeks	Annual	ND
Israel	2000	2012	Infant survey	Children followed from birth to 24 months	‐	50
Italy	2000	2013	National Health survey	Women who gave birth in the 5 years preceding the survey	‐	ND
Latvia	2008	2019	Electronic data (primary health care)	Children 0–12 months	Annual	95
Lithuania	2005	2018	Electronic data (primary health care)	Children 0–12 months	Annual	ND
Luxembourg	2008	2015	Infant survey	Children 4–12 months	7 years	71
Malta	2000	2018	Electronic data (Maternity hospitals)	Birth	Annual	93
Netherlands	2001	2015	Infant survey	Mothers of children under 7 months	^c^	ND
Northern Ireland	2007	2017	Electronic data (primary health care)	Birth to 12 months	Annual	98
Norway	1999	2019	Infant survey	Children 6 and 24 months	6 years	73/47
Poland	2015	2015	Breastfeeding survey	Women who gave birth and followed up to 12 months	‐	100/39/25
Portugal	1996	2014	National Health survey	Women 15–55 years who gave birth	‐	ND
Scotland	2002	2018	Electronic data (primary health care)	Children 0–15 months	‐	70
Spain	1995	2017	National Health survey	Children 0–4 years	‐	72
Sweden	1964	2017	Electronic data (primary health care)	Children 0–12 months	Annual	99
Switzerland	1994	2014	Infant survey	Children 0–12 months	‐	40
Wales	2015	2018	Electronic data (primary health care)	Birth to 12 months	Annual	ND
**Eastern Mediterranean Region**						
Bahrain	1995	2002	Infant survey	Children 0–24 months	‐	ND
Kuwait	2011	2017	Electronic data (primary health care)	Children 0–23 months	7 years	98
Oman, Sultanate	2005	2017	Infant survey	Children 0–24 months	8 years	93
Saudi Arabia	2017	2018	National Health survey	Children under 3 years	‐	ND

Abbreviation: ND, not described.

^a^ According to the last follow‐up.

^b^ Rate refers to the last study follow‐up; more than one rate corresponds to different phases within the study. For electronic data, rate was based on the number of health units that had submitted data or the percent of unknown/missing data reported.

^c^ Not regular.

^d^ Discontinued.

Seventy‐one percent of the countries had updated their indicators since 2015, and 16% have collected breastfeeding indicators for more than two decades. Updates were conducted annually (*n* = 22), every three (*n* = 2) or five to nine (*n* = 6) years. The other 21 countries had no regular updates, or these had been discontinued (Table 2).

Breastfeeding rates were estimated from mothers of children of different age groups: newborns followed up to 8 weeks after birth (*n* = 5), children up to 12 to 15 months (*n* = 17), children under 24 months (*n* = 9) or children under 3 to under 6 years (*n* = 11). In eight surveys, the population sampled was women who had a child in the prior 3 to 5 years (Table 2).

The final response rate was ≥80% in 16 surveys and statistical reports, in contrast with seven with rates below 50% and 13 other sources that did not report this information (Table 2).

### 3.1 Early breastfeeding initiation

Early breastfeeding initiation within the first hour after birth was obtained from nine countries, with the highest rates reported by Brunei Darussalam (92%), Oman (82%) and Luxembourg (71%), and the lowest were Kuwait (39%), Italy (36%) and Korea (32%) (Table S1). Malta and Latvia had data in previous years but discontinued in the updated ones.

Five countries had multiple data points that allowed annual growth estimation. Republic of Korea (4.6 pp), Malta (2.7 pp) and Luxemburg (0.7 pp) showed increases, whereas Latvia (−1.0 pp), Uruguay (−0.2 pp) and Oman (−0.1 pp) reported decreases (Table S2 **)**.

### 3.2 Ever breastfeeding

**FIGURE 1:**
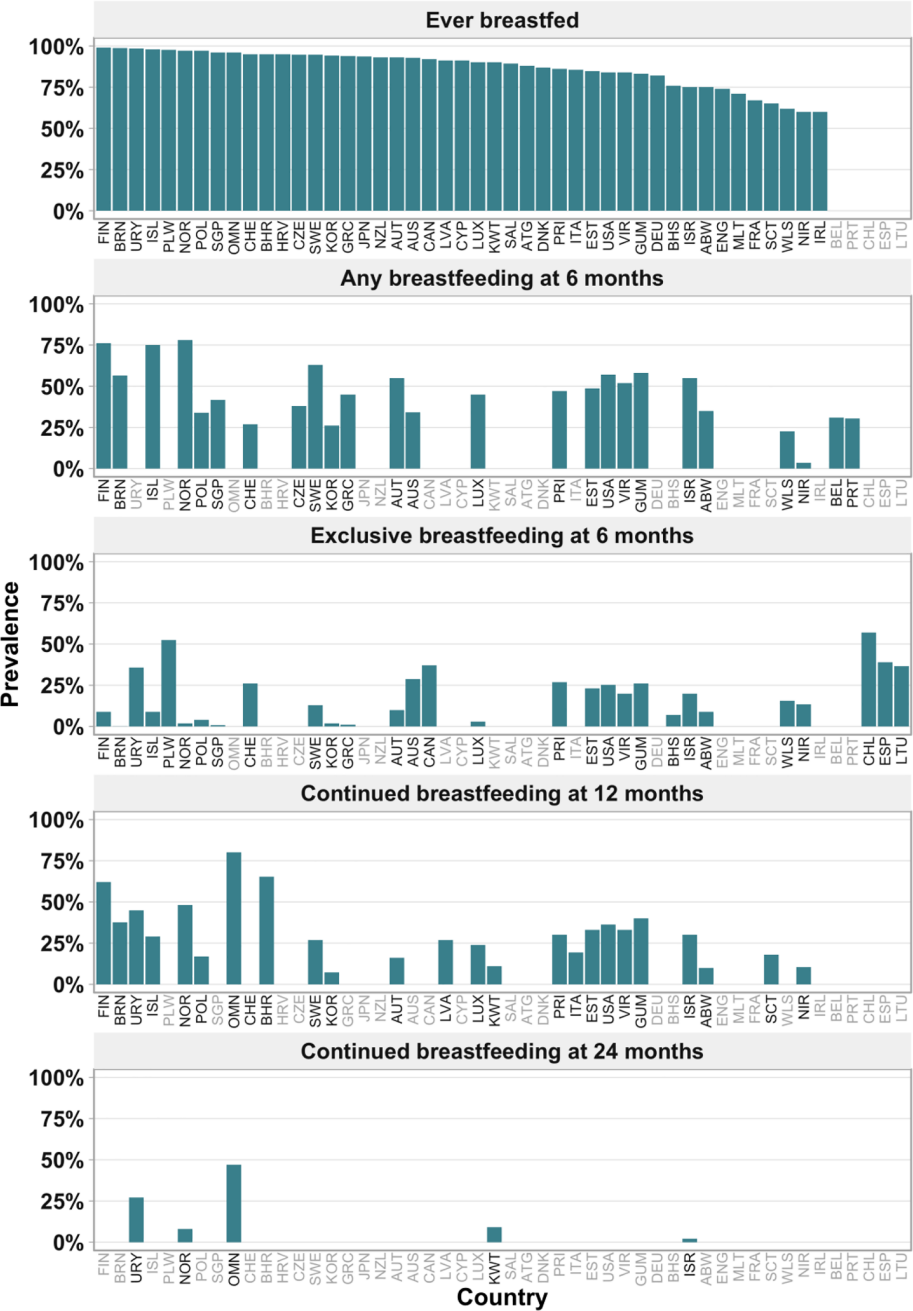
Breastfeeding rates in countries and territories of high‐income countries (*n* = 51) (*Note*. ISO in grey refers to countries with no data in the respective indicator). Abbreviations*:* ATG, Antigua and Barbuda; ABW, Aruba; AUS, Australia; AUT, Austria; BHS, Bahamas; BHR, Bahrain; BEL, Belgium; BRN, Brunei Darussalam; CAN, Canada; CHL, Chile; HRV, Croatia; CYS, Cyprus; CZE, Czech Republic; DNK, Denmark; ENG, England; EST, Estonia; FIN, Finland; FRA, France; DEU, Germany; GRC, Greece; GUM, Guam; ISL, Iceland; IRL, Ireland, Rep; ISR, Israel; ITA, Italy; JPN, Japan; KOR, Korea, Rep; KWT, Kuwait; LVA, Latvia; LTU, Lithuania; LUX, Luxembourg; MLT, Malta; NLD, Netherlands; NZL, New Zealand; NIR, Northern Ireland; NOR, Norway; OMN, Oman; PLW, Palau; POL, Poland; PRT, Portugal; PRI, Puerto Rico; SAL, Saudi Arabia; SCT, Scotland; SGP, Singapore; ESP, Spain; SWE, Sweden; CHE, Switzerland; USA, United States; URY, Uruguay; VIR, Virgin Islands; WLS, Wales

Forty‐six countries presented data on the prevalence of ever breastfed children, with a median of 91%. Twenty‐three countries reported a prevalence higher than 90%, 13 countries reported a prevalence between 80% and 90%, and 10 reported a prevalence between 60% and 79%. The lowest prevalence levels were reported in Scotland (65%), Wales (62%), Northern Ireland (60%) and the Republic of Ireland (60%) (Figure 1, Table S1).

**FIGURE 2:**
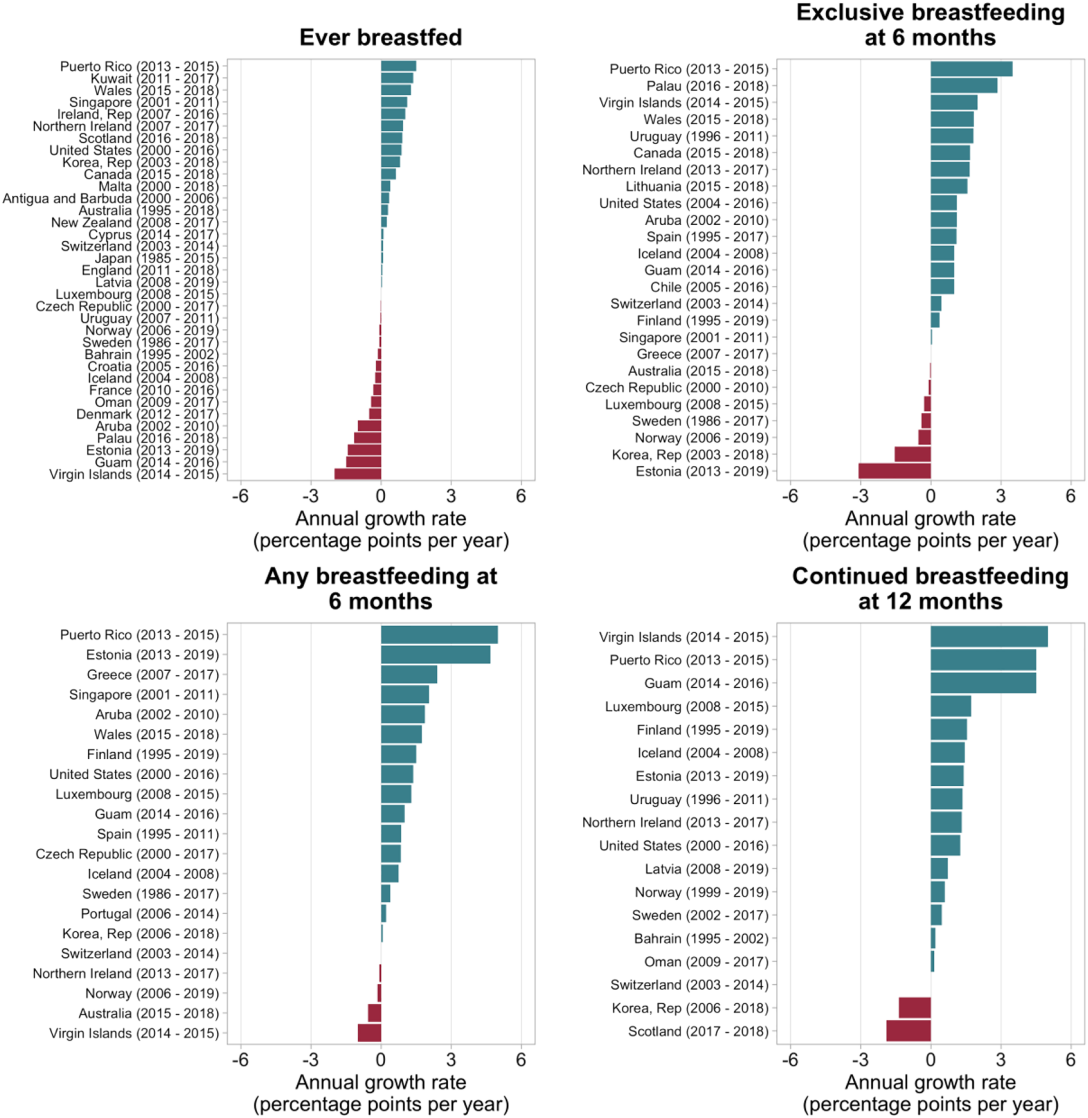
Breastfeeding annual growth rates of high‐income countries with available time series data

The annual growth rate for ever breastfed children varied from 1.5 pp to −2.0 per year, with positive changes in half of the countries with data. The highest growth rates were found in Puerto Rico (1.5 pp), Kuwait (1.4 pp) and Wales (1.3 pp), and the lowest were found in the Virgin Islands (−2.0 pp), Guam (−1.5 pp) and Estonia (−1.4 pp) (Figure 2, Table S2). There was no significant trend (*P* = 0.273) in this indicator over time, with median values of 90% in the earlier and 91% in the most recent estimate (data not shown in tables).

### 3.3 Exclusive breastfeeding under 6 months

Forty countries presented at least one indicator of exclusive breastfeeding at a given age under 6 months. Data availability according to time frame varied widely among countries. Of the 20 countries with data at the age of 3 months, the highest rates were reported by Uruguay (72%), Iceland (69%) and Norway (68%), and the lowest were Czech Republic (21%), Northern Ireland (19%) and Aruba (12%) (Table 3). The annual growth was estimated for 16 countries with changes varying from 6.5 pp to −3.0 pp. The highest growth rates were observed for Puerto Rico (6.5 pp), Guam (3.0 pp) and Uruguay (2.7 pp), and the lowest for the Virgin Island (−3.0 pp), Czech Republic (−1.9 pp) and Estonia (−0.3 pp) (Table S2).

**TABLE 3:** Exclusive breastfeeding rates available from high‐income countries (*n* = 40)

Country/territories	Year	Exclusive breastfeeding									
		1 week	2 weeks	1 month	2 months	3 months	0–3 months	4 months	5 months	0–5 months	6 months
**Region of the Americas**											
Aruba	2010			21	13	12		11	10		9
Bahamas	2001										7
Canada	2018										37
Chile	2016										57
Puerto Rico	2015					48					27
United States	2016					48					25
Uruguay	2011			89	77	72		66	49	65	36
Virgin Islands	2015					32					20
**Western Pacific Region**											
Australia	2018				71			58			29
Brunei Darussalam	2009			68	51	40		33	27		0.1
Guam	2016					47					26
Korea, Republic	2018	16	37	37	35	31		26	15		2
New Zealand	2017		78								
Palau	2018										52
Singapore	2011			35	28						0.8
**European Region**											
Austria	2006					60					10
Croatia, Republic	2016				68^c^				62^h^		
Czech Republic	2017			33^a^		21					
Estonia	2019			78		65					23
Finland	2019			58	54	53		50	26	60	9
Greece	2017	51		40				25			0.8
Iceland	2008	85		76^a^		69			38		9
Israel	2012				58						20
Italy	2013			49		44^f^			39^i^	43	
Latvia	2019			45^b^			34			19	
Lithuania	2018					53					37
Luxembourg	2015							33			3
Netherlands	2015	64	59	57	53	47		45	42		39
Northern Ireland	2017			22^a^		19					14
Norway	2019	87	85	81	74	68		39	13		2
Poland	2015				44			29			4
Portugal	2014					56		49			
Scotland	2018				32^d^						
Spain	2017			74^a^		64					39
Sweden	2017	75			62			50			13
Switzerland	2014				71^e^			62^g^			26^j^
Wales	2018			25^a^							16
**Eastern Mediterranean Region**											
Kuwait	2017						10			8	
Oman	2017									23	
Saudi Arabia	2018									48	
*Total of indicators*	*6*	*4*	*17*	*15*	*20*	*2*	*14*	*10*	*7*	*30*	
*Median*	*70*	*69*	*49*	*54*	*48*	*22*	*42*	*33*	*43*	*18*	
*Min–Max*	*16–87*	*37–85*	*21–89*	*13–77*	*12–72*	*10–34*	*11–66*	*10–62*	*8–65*	*0.1–57*	

^a^ 6 weeks.

^b^ <6 weeks.

^c^ 0–2 months.

^d^ 6–8 weeks.

^e^ 1–2 months.

^f^ 2–3 months.

^g^ 3–4 months.

^h^ 3–5 months.

^i^ 4–5 months.

^j^ 5–6 months.

Fourteen countries reported exclusive breastfeeding at the age of 4 months. The highest rates were reported by Uruguay (66%), Switzerland (62%) and Australia (58%), and the lowest were reported by Republic of Korea (26%), Greece (25%) and Aruba (11%) (Table 3). Eight out of 14 countries presented multiple data to estimate the annual growth, with changes varying from 3.0 pp to −0.5 pp. The highest annual growth rates were reported by Finland (3.0 pp), Uruguay (2.9 pp), Portugal (0.9 pp) and Aruba (0.9 pp) (Table S2 **)**.

### 3.4 Exclusive breastfeeding at 6 months

Thirty countries presented data on exclusive breastfeeding at 6 months with levels varying from 0.1% to 57%, with a median of 18%. The highest values were reported for Chile (57%), Palau (52%), Netherlands (39%) and Spain (39%), whereas the lowest were reported for Republic of Korea (2%), Norway (2%), Greece and Singapore (0.8%) and Brunei Darussalam (0.1%) (Figure 1, Table 3).

Regarding annual growth for exclusive breastfeeding at 6 months, 17 out of 25 countries with two points in time presented positive values ranging from 3.5 pp to 0.1 pp. Puerto Rico (3.5 pp), Palau (2.5 pp), Virgin Islands (2.0 pp), Uruguay (1.8 pp) and Wales (1.8 pp) presented the highest increases, whereas Estonia (−3.6 pp) and Republic of Korea (−1.6 pp) presented the highest decreases (Figure 2, Table S2). Taking all countries together, there was a significant increase over time (*P* = 0.061 in the Wilcoxon test) at the level 10%, with median values of 18% and 23% in the earlier and later measurements (data not shown in tables).

### 3.5 Any breastfeeding at 6 months

Any breastfeeding at 6 months was reported by 21 countries. The prevalence varied from 4% to 78% among countries, with a median of 45%. Rates above 50% were reported by Norway (78%), Finland (76%), Iceland (75%), Sweden (63%), Guam (58%), Brunei Darussalam (57%), the United States (57%), Austria (55%), Israel (55%) and the Virgin Islands (52%). The lowest prevalence levels were reported by Republic of Korea (26%), Wales (23%) and Northern Ireland (4%) (Figure 1, Table S1).

Sixteen out of 21 countries presented an annual increase, ranging from 5.5 pp to 0.1 pp, and Puerto Rico (5.0 pp), Estonia (4.7 pp) and Greece (2.4 pp) presented the highest annual increase rates. Four countries presented declines ranging from −0.1 pp to −1.0 pp; the Virgin Islands (−1.0 pp) and Australia (−0.6 pp) presented the highest decrease rates (Figure 2, Table S2). In the group of countries with two measurements over time, the median value increased significantly from 33% to 45% (*P* = 0.0003 in the Wilcoxon test).

### 3.6 Continued breastfeeding at 12 months

Twenty‐five countries reported data on continued breastfeeding at 12 months with a median value of 29%. Aruba (10%), Republic of Korea (7%) and Switzerland (0%) presented the lowest levels, whereas the highest levels were reported by Oman (80%), Bahrain (65%) and Norway (48%) (Figure 1, Table S1).

Annual changes in continued breastfeeding at 12 months were estimated for 18 countries, with increases observed in 15 countries. The highest increases were in the Virgin Islands (5.0 pp), Puerto Rico (4.5 pp) and Guam (4.5 pp), whereas decreases were observed in Scotland (−1.9 pp) and Republic of Korea (−1.4 pp) (Figure 2,Table S2). For the 18 countries with two measures over time, the median value increased significantly from 24% to 32% (*P* = 0.002 in the Wilcoxon test) (data not shown in tables).

### 3.7 Continued breastfeeding at 24 months

Continued breastfeeding at 24 months was only reported by five countries. Oman (47%) and Uruguay (27%) presented the highest prevalence with annual increases of 0.4 pp and 1.0 pp per year, respectively. In contrast, Kuwait (9%), Norway (8%) and Israel (2%) reported prevalence levels below 10% (Figure 1, Tables S1 and S2).

### 3.8 Annual changes within countries with conflicting trends

The annual changes in breastfeeding rates were not consistent within some countries, in which an observed increase in one indicator contrasted with a decrease in others. Although the Virgin Islands and Guam showed the largest declines (−2.0 pp to −1.5 pp) in the ever‐breastfed indicator, both presented the highest increases in continued breastfeeding at 12 months. Estonia showed decreases in ever breastfeeding (−1.4 pp) and exclusive breastfeeding at 6 months (−3.1 pp) but a substantial increase in any breastfeeding at 6 months (4.7 pp) and at 12 months (1.4 pp). In contrast, Republic of Korea, which presented an increase in ever breastfeeding (0.8 pp), showed an important decline in exclusive breastfeeding at 6 months (−1.6 pp) and continued breastfeeding at 12 months (−1.4 pp) (Figure 2, Table S2).

## 4 DISCUSSION

We described the current patterns and annual growth in breastfeeding rates in high‐income national economies, with emphasis on the strengths and weaknesses of available data. Using different sources of data, we were able to obtain at least one breastfeeding indicator for 51 countries, with recent estimates and time trends for most of these countries. Nevertheless, the lack of standardized methodologies and definitions affected the comparability of breastfeeding indicators, especially related to the variability of the time frame used to characterize exclusive breastfeeding under 6 months. The present analyses provide the basis for future studies comparing national recommendations and actual practices.

Our data confirm that most children (median of 91%) in high‐income countries are ever breastfed, but there are marked declines in the first months of life. By 6 months of age, the median prevalence equals 18% for exclusive and 45% for any breastfeeding. The number of countries that monitor breastfeeding indicators from 6 months onwards decreases by one third. The lack of data suggests that continued practice of breastfeeding after 6 months does not seem to be strongly encouraged in high‐income countries, as these data are being monitored by a few countries. It may also reflect the fact that longer durations of breastfeeding are not sufficiently valued in several high‐income countries.

Most countries have adopted the WHO and United Nations Children's Fund (UNICEF) recommendation of exclusive breastfeeding for 6 months, have initiatives to support breastfeeding and attempt to monitor trends. Nevertheless, our review observed some contradictions regarding recommendations on the duration of exclusive breastfeeding. The European Society for Paediatric Gastroenterology, Hepatology, and Nutrition Committee on Nutrition supports the recommendation of exclusive breastfeeding for the first 6 months, yet advises that complementary feeding should not be introduced before 4 months nor be delayed beyond 26 weeks (6 months) (Fewtrell et al., 2017). Consequently, in most European countries, solid food is introduced before the age of 6 months (Carletti, Pani, Monasta, Knowles, & Cattaneo, 2017; Tromp et al., 2013). We did not attempt to document national recommendations in all countries included in our review, but one possible explanation for the lack of consistency with internationally recommended breastfeeding indicators may be that national recommendations may vary from country to country.

Another relevant aspect in the comparison of exclusive breastfeeding levels relies on that a few high income countries report on this indicator according to the WHO definition, i.e., the proportion of children 0–5 months who were fed exclusively with breast milk, using standardized questions based on 24‐h recall––such as those applied in nationally representative surveys carried out in low and middle income countries (LMIC), including Demographic Health Survey and Multiple Indicator Cluster Surveys. Lack of standardization limits the number of high‐income countries with comparable statistics with LMIC to conduct further analysis of global trends of exclusive breastfeeding.

Despite data limitations, it was possible to calculate time trends for most countries for at least one indicator. Of the 36 countries with trend data on ever breastfed, 17 showed increases over time. For exclusively breastfeeding at 6 months, 17 of 25 countries showed increased breastfeeding, and the corresponding numbers for any breastfeeding at 6 months were increased in 16 of 21 countries and for continued breastfeeding at 12 months in 15 out of 18 countries. This suggests that breastfeeding practices are improving in most high‐income countries that have monitored their indicators. A caveat of these analyses is that few countries report on standard errors for the observed estimates, so it is not possible to assess whether the changes over time were statistically significant. Conflicting patterns in annual growth for different indicators, which were observed within some countries, may suggest that programmes and policies have not uniformly targeted all stages of the first year of the child's life. For example, maintenance of exclusive breastfeeding up to 6 months and of continued breastfeeding for up to 12 months require specific interventions (Rollins et al., 2016). Initiatives to support breastfeeding must consider its multifactorial determinants, which include individual factors, social attitudes and values, workplace protection, maternity leave legislation and enforcement, control of advertising by formula companies and health care services that support breastfeeding. Programmes and interventions must target both antenatal and post‐natal periods, be extended to mothers, fathers and family members and be combined with the relevant policies and regulations mentioned above (Rollins et al., 2016). Assessment of breastfeeding programmes and monitoring standardized indicators every 5 years are also essential activities (WHO & UNICEF, 2019).

National data come from population‐based sample surveys, health reports from electronic data reported by maternity hospitals and medical records of primary care services. Concerning data from national surveys, methodological issues arise from the reliance on variable data collection strategies. Studies with data collected through mail questionnaires or telephone usually result in low response rates, often below 50%. An exception was observed in Luxembourg, where response rates to mailed questionnaires improved from 59% in 2008 to 71% in 2015. Overall, few surveys discuss the methodological challenges associated with low response rates. The Centers for Disease Control and Prevention that monitor breastfeeding data of the United States, the Virgin Islands and Puerto Rico adopt the strategy of adding breastfeeding questions in national immunization surveys applied by telephone. A noteworthy change is that since 2018, the sampling frame was restricted to cell phones, whereas landlines were also used prior, and the response rate dropped to 33%. Thus, the lack of methodological standardization, especially in the study design and data collection strategy, may result in marked differences in representativeness and response rates among surveys within the same country. Nevertheless, there have been improvements in data collection strategies to estimate breastfeeding indicators in some countries such as Australia, Italy, Finland and Oman. Australia and Italy made methodological improvements in the last round of data collection, although this affected the comparability with indicators estimated in earlier surveys. Oman and Finland have adopted the use of standard WHO indicators in their recent surveys. These four countries show that, with sufficient motivation at the national level, high‐income countries can produce standardized information on breastfeeding that is comparable to what is already available for most low and middle‐income countries.

Some countries have replaced surveys with electronic medical records resulting in national censuses of children followed in primary care services or maternity hospitals. The United Kingdom has discontinued the unified Infant Feeding Survey after 2010 (UK Government & Department of Health, 2019), and breastfeeding rates have been summarized using electronic data through the National Health System (NHS) independently in England, Wales, Ireland and Northern Ireland onwards; however, the NHS system is limited to breastfeeding indicators up to 8 weeks. Nevertheless, Northern Ireland has linked maternity hospital registry data to primary care services, allowing complete follow‐up from birth to 12 months, and the estimation of six indicators with the inclusion of exclusive breastfeeding rates since 2014 (Department of Public Health & Public Health Agency, 2020), and Scotland has combined surveys of newborn first Health Visitors review and child health immunization programmes up to 13 to 15 months of age (Scottish Government, Health and Social Care & NHS Information Services Division, 2019).

The longest history of use of electronic data is Sweden, which has pooled national data from primary care facilities, with available estimates for exclusive breastfeeding since 1964, and for any breastfeeding from the age of 1 week to 12 months since 1986 (Sweden National Board of Health and Welfare, 2019). Despite being an interesting initiative for countries without regular surveys, caution must be observed in the attempt to use data gathered from electronic records: the national electronic system must be uniform and cover all health care units, information must be aligned with WHO definitions, health care workers must be trained and data submission must be mandatory for all health care units (Bagci Bosi et al., 2016). Furthermore, health reports must provide complete methodological documentation of breastfeeding questions, indicator definitions, number of censored children, standard errors for the estimates and missing data. In the present work, few electronic data presented a complete methodological report, which required our team to contact the research institutions to obtain such information. Some of these limitations were addressed by Milos, Rodin, Tjesic‐Drinkovic, and Mujkic (2019) in their attempt to use electronic data from Croatia (Milos et al., 2019). In their case, because breastfeeding information was not collected at similar ages for all children, the database only allowed for the estimation of indicators in broad age categories, and in addition, the percentage of unreported data fluctuated in specific years and imposed difficulties in drawing time trends.

Some difficulties were faced during our search process. Some reports are published in their native language, which make them difficult to identify using our search strategies, as well as hindering their use by the global scientific community. Although we have followed a broad protocol for searching the grey literature, in some countries, this failed to produce any information, but existing data reports were identified after contacting institutions and researchers through e‐mail. Additionally, some reports lacked details on important methodological aspects, such as description of breastfeeding definitions, target population and sample response rate. In three cases, raw data were obtained from national sources, and we were able to calculate the indicators. While a number of countries stand out for their high quality, methodologically sound reports (Australia, Luxembourg, Norway, Finland, Northern Ireland and Oman), the available reports for other countries (such as Antigua and Barbuda, Aruba, Saudi Arabia, Portugal and Spain) were not very informative.

The strengths of our review include the ability to obtain updated information on breastfeeding indicators for a substantial number of countries without standardized surveys due to our comprehensive search of different data types. In addition, we were able to estimate time trends for most indicators. The main challenge of the current review was the lack of regular surveys in most countries and difficulties in identifying existing data sources in some countries. For example, we were unable to find any data for 31 high‐income countries and territories. In addition, breastfeeding data were derived from a variety of sources, and the age of measurement often failed to agree with the international definitions, which limited between‐country comparisons. Lack of uniformity was particularly common for exclusive breastfeeding indicators. As a consequence, it was not possible to stratify our analyses based on methodology (study design, population and age definitions), source of data (electronic versus survey) and final response rates.

## 5 CONCLUSIONS

Our review shows that current breastfeeding practices in most high‐income countries fall well short of international recommendations of exclusive breastfeeding for 6 months and continued breastfeeding until 2 years. Our analyses of the median values of countries with available data show that although nine out of 10 children start to be breastfed, only about half are still breastfed (and one quarter exclusively breastfed) at 6 months, and one third remain on the breast at 12 months. On the positive side, there were significant increases in all indicators under study, except for ever breastfeeding. Stronger interventions are needed to promote breastfeeding in high‐income countries as a whole, and investments are required to monitor trends with standardized methodologies.

## CONFLICTS OF INTEREST

The authors declare that they have no conflicts of interest.

## CONTRIBUTIONS

C‐V formulated the research question. JSV and MFSM performed the data search. TMS and LPV‐ provided statistical and graphical support. JSV and CV wrote the article. PARN, TMS and LPV‐ provided critical revision.

## Supporting information


**Table S1.** Complete table with most updated breastfeeding rates available from high‐income countries (n = 51).
**Table S2.** Annual growth rate of breastfeeding indicators of high‐income countries and territories with comparable estimations across years.^1^

## APPENDIX A

### Major scientific databases (published peer‐reviewed literature)

The following search strategy was used in PubMed ^1^
Available from: www.pubmed.com. :

(Andorra OR Antigua and Barbuda OR Argentina OR Aruba OR Australia OR Austria OR Bahamas OR Bahrain OR Barbados OR Belgium OR Bermuda OR “British Virgin Islands” OR “Brunei Darussalam” OR Canada OR “Cayman Islands” OR “Channel Islands” OR Chile OR Curacao OR Cyprus OR “Czech Republic” OR Denmark OR England OR Estonia OR “Faroe Islands” OR Finland OR France OR “French Polynesia” OR Germany OR Gibraltar OR Greece OR Greenland OR Guam OR “Hong Kong” OR Hungary OR Iceland OR Ireland OR “Isle of Man” OR Israel OR Italy OR Japan OR Korea OR Kuwait OR Latvia OR Liechtenstein OR Lithuania OR Luxembourg OR “Macao” OR Malta OR Monaco OR Netherlands OR “New Caledonia” OR “New Zealand” OR “Northern Mariana Islands” OR Norway OR Oman OR Palao OR Poland OR Portugal OR “Puerto Rico” OR Qatar OR “San Marino” OR “Saudi Arabia” OR Scotland OR Seychelles OR Singapore OR “Sint Maarten” OR “Slovak Republic” OR Slovenia OR Spain OR “Saint Kitts and Nevis” OR “St Martin” OR Sweden OR Switzerland OR “Trinidad and Tobago” “Turks and Caicos Islands” OR “United Arab Emirates” OR “United Kingdom” OR “United States” OR Uruguay OR “Virgin Islands”)

AND

(breastfeeding) OR (breast feeding) OR (breastfeeding practices) OR (breastfed) OR (breastfeed) OR (infant feeding) OR (infant feeding practices)

AND

(National Survey) OR (National Health) OR (Nutrition Survey) OR (Nutritional Survey) OR (Nutritional Surveys) OR (Health Survey) OR (Surveys Health)

A total of 2,806 references were identified as a result of this search.

### Grey literature

The following search strategy is being used in Google (results not yet compiled):

Country name + ((breastfeeding) OR (breast feeding) OR (breastfeeding practices) OR (breastfed) OR (breastfeed) OR (infant feeding) OR (infant feeding practices)) + site:.gov
Country name + ((breastfeeding) OR (breast feeding) OR (breastfeeding practices) OR (breastfed) OR (breastfeed) OR (infant feeding) OR (infant feeding practices)) + file:.pdf
Country name + ((breastfeeding) OR (breast feeding) OR (breastfeeding practices) OR (breastfed) OR (breastfeed) OR (infant feeding) OR (infant feeding practices)) + file:.xls
Country name + allintitle:((breastfeeding) OR (breast feeding) OR (breastfeeding practices) OR (breastfed) OR (breastfeed) OR (infant feeding) OR (infant feeding practices))
Country name + allintext:((breastfeeding) OR (breast feeding) OR (breastfeeding practices) OR (breastfed) OR (breastfeed) OR (infant feeding) OR (infant feeding practices))

Other grey literature searches are also being reviewed:

○ Ministries of health of all countries
○ WORLDCAT: www.worldcat.org
○ STATISTICS OFFICES: https://globaledge.msu.edu/globalresources/resourcesbytag/statistics‐office
○ PACIFIC ISLAND COUNTRIES AND TERRITORIES: http://prism.spc.int/component/finder/search?q=health&Itemid=262
○ COUNTRY PLANNING CYCLE: http://www.nationalplanningcycles.org/file‐repository/CAF

## APPENDIX B

Country, source and most updated year with breastfeeding indicator data (*n* = 51)

**Table:**

Country/territories	Data source	Year
Antigua and Barbuda	Living Conditions and Household Budget Survey	2006
Aruba	Health Monitor 2013 Aruba	2010
Australia	National Aboriginal and Torres Strait Islander Social Survey & Australia National Health Survey	2018
Austria	Säuglingsernährung Heute (Infant nutrition today)	2006
Bahamas	Bahamas Living Conditions Survey	2001
Bahrain	Breastfeeding Patterns & Practices in the Kingdom of Bahrain (2002)	2002
Belgium	Vaccination coverage survey in infants and adolescent	2012
Brunei Darussalam	National Health and Nutritional Status Survey	2009
Canada	Canadian Community Health Survey	2018
Chile	Vigilancia del Estado Nutricional de la Población Bajo Control y de la Lactancia Materna	2016
Croatia, Republic	Public Health Reports from Primary Health Care Services and Maternity wards	2016
Cyprus	Cyprus Public and Private Maternity Units	2017
Czech Republic	Institute of Health Information and Statistics	2017
Denmark	Den Nationale Børnedatabase	2017
England	NHS Hospital Episode Statistics for England	2018
Estonia	Tervisestatistika ja Terviseuuringute	2019
Finland	Imeväisikäisten ruokinta Suomessa (Infant Feeding Survey)	2019
France	Enquête Nationale Périnatale Rapport	2016
Germany	Kinder‐und Jugendgesundheitssurvey ‐ KiGGS (Welle 1)	2012
Greece	Εθνική Μελέτη εκτίμησης της συχνότητας και των προσδιοριστικών παραγόντων του Μητρικού Θηλασμού στην Ελλάδα	2017
Guam	National Immunization Survey. Center for Disease Control and Prevention	2016
Iceland	Brjóstagjöf og næring ungbarna á Íslandi sem fædd eru	2008
Ireland, Republic	National Perinatal Reporting System	2016
Israel	סקר בריאות ותזונה לאומי (National Health and Nutrition Survey from birth until age 2 years)	2012
Italy	Gravidanza, Parto e Allatamento al Seno ‐ Condizioni di Salute e Ricorso ai Servizi Sanitari	2013
Japan	乳幼児栄養調査 (Infant Feeding Survey)	2015
Korea, Republic	한국의 출산 및 가족 건강 및 복지에 관한 전국 조사 (National Survey on Fertility and Family Health and Welfare in Korea)	2018
Kuwait	(Kuwait Nutritional Surveillance System) نظام الكويت للمراقبة الغذائية	2017
Latvia	Veselības statistikas datubāze (Health Statistics Database)	2019
Lithuania	Metinių sveikatos statistikos ataskaitų suvestinės (Summaries of annual health statistics reports)	2018
Luxembourg	Peiling melkvoeding van zuigelingen (Infant Feeding Survey)	2015
Malta	National Obstetric Information System	2018
Netherlands	Peiling melkvoeding van zuigelingen (Infant Feeding Survey)	2015
New Zealand	Report on Maternity	2017
Northern Ireland	Northern Ireland Maternity System and Northern Ireland Child Health System	2017
Norway	Spedkost 3‐6, 12 & 24 måneder	2019
Oman, Sultanate	Oman National Nutrition Survey	2017
Palau	Maternal na Child Health Services Annual Report	2018
Poland	Breastfeeding Newborns and Infants up to 12 Months of Life	2015
Portugal	Inquéritos Nacionais de Saúde	2014
Puerto Rico	National Immunization Survey. Center for Disease Control and Prevention	2015
Saudi Arabia	(Household Health Survey) مسح†صحة†الأسرة	2018
Scotland	Scottish Maternity Care Survey. Infant Feeding Statistics	2018
Singapore	National Breastfeeding Survey	2011
Spain	Encuesta Nacional de Salud	2017
Sweden	National Board of Health and Welfare	2017
Switzerland	Swiss Infant Feeding Study	2014
United States	National Immunization Survey. Center for Disease Control and Prevention	2016
Uruguay	Encuesta de Lactancia Estado Nutricional y Alimentación complementaria	2011
Virgin Islands	National Immunization Survey. Center for Disease Control and Prevention	2015
Wales	National Community Child Health Database. NHS Wales Informatics Service	2018
