# Supplementary material for: Monitoring breastfeeding indicators in high‐income countries: Levels, trends and challenges
Source: Matern Child Nutr. 2021 Jan 6;17(3):e13137. doi: 10.1111/mcn.13137 (PMC8189208; doi:10.1111/mcn.13137)
Supplement: Supplementary file 1 — Table S1. Complete table with most updated breastfeeding rates available from high‐income countries (n = 51). Table S2. Annual growth rate of breastfeeding indicators of high‐income countries and territories with comparable estimations across years.1 [file MCN-17-e13137-s001.docx]

Table S1. Complete table with most updated breastfeeding rates available from high-income countries (n=51).

|  |  | **BREASTFEEDING INDICATORS** | | | | | | | | | | | | | | |  |
| --- | --- | --- | --- | --- | --- | --- | --- | --- | --- | --- | --- | --- | --- | --- | --- | --- | --- |
|  |  |  |  | **Exclusive breastfeeding** | | | | | | | | | |  |  |  |  |
| **Country/territories** | **Year** | **Early initiation (1hr)** | **Ever breastfed**† | **1-wk** | **2-wks** | **1-month** | **2 months** | **3 months** | **0-3 months** | **4 months** | **5 months** | **0-5 months** | **6 months** | **Any 6 months** | **Continued 12 months** | **Continued 24 months** | **N*** |
| **Region of the Americas** | | |  |  |  |  |  |  |  |  |  |  |  |  |  |  |  |
| Antigua and Barbuda | 2006 |  | 88(a) |  |  |  |  |  |  |  |  |  |  |  |  |  | 1 |
| Aruba | 2010 |  | 75(b) |  |  | 21 | 13 | 12 |  | 11 | 10 |  | 9 | 35 | 10 |  | 9 |
| Bahamas | 2001 |  | 76 |  |  |  |  |  |  |  |  |  | 7 |  |  |  | 2 |
| Canada | 2018 |  | 92 |  |  |  |  |  |  |  |  |  | 37 |  |  |  | 2 |
| Chile | 2016 |  |  |  |  |  |  |  |  |  |  |  | 57 |  |  |  | 1 |
| Puerto Rico | 2015 |  | 86 |  |  |  |  | 48 |  |  |  |  | 27 | 47 | 30 |  | 5 |
| United States | 2016 |  | 84 |  |  |  |  | 48 |  |  |  |  | 25 | 57 | 36 |  | 5 |
| Uruguay | 2011 | 60 | 98 |  |  | 89 | 77 | 72 |  | 66 | 49 | 65 | 36 |  | 45(x) | 27(w) | 11 |
| Virgin Islands | 2015 |  | 84 |  |  |  |  | 32 |  |  |  |  | 20 | 52 | 33 |  | 5 |
| **Western Pacific Region** | | |  |  |  |  |  |  |  |  |  |  |  |  |  |  |  |
| Australia | 2018 |  | 93 |  |  |  | 71 |  |  | 58 |  |  | 29 | 34 |  |  | 5 |
| Brunei Darussalam | 2009 | 92 | 99 |  |  | 68 | 51 | 40 |  | 33 | 27 |  | 0.1 | 57 | 38 |  | 10 |
| Guam | 2016 |  | 83 |  |  |  |  | 47 |  |  |  |  | 26(s) | 58 | 40 |  | 5 |
| Japan | 2015 |  | 94(b) |  |  |  |  |  |  |  |  |  |  |  |  |  | 1 |
| Korea. Republic | 2018 | 32 | 94 | 16 | 37 | 37 | 35 | 31 |  | 26 | 15 |  | 2 | 26 | 7 |  | 12 |
| New Zealand | 2017 |  | 93 |  | 78 |  |  |  |  |  |  |  |  |  |  |  | 2 |
| Palau | 2018 |  | 98 |  |  |  |  |  |  |  |  |  | 52 |  |  |  | 2 |
| Singapore | 2011 |  | 96 |  |  | 35 | 28 |  |  |  |  |  | 0.8 | 42 |  |  | 5 |
| **European Region** | | |  |  |  |  |  |  |  |  |  |  |  |  |  |  |  |
| Austria | 2006 |  | 93 |  |  |  |  | 60 |  |  |  |  | 10 | 55 | 16 |  | 5 |
| Belgium | 2012 |  |  |  |  |  |  |  |  |  |  |  |  | 31 |  |  | 1 |
| Croatia. Republic | 2016 |  | 95 |  |  |  | 68(g) |  |  |  | 62(p) |  |  |  |  |  | 3 |
| Cyprus | 2017 |  | 91 |  |  |  |  |  |  |  |  |  |  |  |  |  | 1 |
| Czech Republic | 2017 |  | 95 |  |  | 33(e) |  | 21 |  |  |  |  |  | 38 |  |  | 4 |
| Denmark | 2017 |  | 87(‡) |  |  |  |  |  |  |  |  |  |  |  |  |  | 1 |
| England | 2018 |  | 74 |  |  |  |  |  |  |  |  |  |  |  |  |  | 1 |
| Estonia | 2019 |  | 85 |  |  | 78 |  | 65 |  |  |  |  | 23 | 49 | 33 |  | 6 |
| Finland | 2019 |  | 99 |  |  | 58 | 54 | 53 |  | 50 | 26 | 60 | 9 | 76 | 62(t) |  | 10 |

*continue*

|  |  |  |  | **Exclusive breastfeeding** | | | | | | | | | |  |  |  |  |
| --- | --- | --- | --- | --- | --- | --- | --- | --- | --- | --- | --- | --- | --- | --- | --- | --- | --- |
| **Country/territories** | **Year** | **Early initiation (1hr)** | **Ever breastfed**† | **1-wk** | **2-wks** | **1-month** | **2 months** | **3 months** | **0-3 months** | **4 months** | **5 months** | **0-5 months** | **6 months** | **Any 6 months** | **Continued 12 months** | **Continued 24 months** | **N*** |
| France | 2016 |  | 67 |  |  |  |  |  |  |  |  |  |  |  |  |  | 1 |
| Germany | 2012 |  | 82 |  |  |  |  |  |  |  |  |  |  |  |  |  | 1 |
| Greece | 2017 |  | 94 | 51 |  | 40 |  |  |  | 25 |  |  | 0.8 | 45 |  |  | 6 |
| Iceland | 2008 |  | 98 | 85 |  | 76(e) |  | 69 |  |  | 38 |  | 9 | 75 | 29 |  | 8 |
| Ireland. Republic | 2016 |  | 60 |  |  |  |  |  |  |  |  |  |  |  |  |  | 1 |
| Israel | 2012 | 41 | 75(c) |  |  |  | 58 |  |  |  |  |  | 20 | 55 | 30 | 2 | 7 |
| Italy | 2013 | 36 | 86 |  |  | 49 |  | 44(m) |  |  | 39(q) | 43 |  |  | 19(x) |  | 7 |
| Latvia | 2019 |  | 91(d) |  |  | 45(f) |  |  | 34 |  |  | 19 |  |  | 27(r) |  | 5 |
| Lithuania | 2018 |  |  |  |  |  |  | 53 |  |  |  |  | 37 |  |  |  | 2 |
| Luxembourg | 2015 | 71 | 90 |  |  |  |  |  |  | 33 |  |  | 3 | 45 | 24 |  | 6 |
| Malta | 2018 |  | 71 |  |  |  |  |  |  |  |  |  |  |  |  |  | 1 |
| Netherlands | 2015 |  | 80 | 64 | 59 | 57 | 53 | 47 |  | 45 | 42 |  | 39 | 32 |  |  | 10 |
| Northern Ireland | 2017 |  | 60 |  |  | 22(e) |  | 19 |  |  |  |  | 14 | 4 | 11 |  | 6 |
| Norway | 2019 |  | 97 | 87 | 85 | 81 | 74 | 68 |  | 39 | 13 |  | 2 | 78 | 48 | 8 | 12 |
| Poland | 2015 |  | 97 |  |  |  | 44 |  |  | 29 |  |  | 4 | 34 | 17(x) |  | 6 |
| Portugal | 2014 |  |  |  |  |  |  | 56 |  | 49 |  |  |  | 30 |  |  | 3 |
| Scotland | 2018 |  | 65 |  |  |  | 32(h) |  |  |  |  |  |  |  | 18(z) |  | 3 |
| Spain | 2017 |  |  |  |  | 74(e) |  | 64 |  |  |  |  | 39 |  |  |  | 3 |
| Sweden | 2017 |  | 95 | 75 |  |  | 62 |  |  | 50 |  |  | 13 | 63 | 27 |  | 7 |
| Switzerland | 2014 | 67 | 95 |  |  |  | 71(i) |  |  | 62(n) |  |  | 26(s) | 27 | 0(v) |  | 7 |
| Wales | 2018 |  | 62 |  |  | 25(e) |  |  |  |  |  |  | 16 | 23 |  |  | 4 |
| **Eastern Mediterranean Region** | | |  |  |  |  |  |  |  |  |  |  |  |  |  |  |  |
| Bahrain | 2002 |  | 95 |  |  |  |  |  |  |  |  |  |  |  | 65 |  | 2 |
| Kuwait | 2017 | 39 | 90 |  |  |  |  |  | 10 |  |  | 8 |  |  | 11 | 9 | 6 |
| Oman | 2017 | 82 | 96 |  |  |  |  |  |  |  |  | 23 |  |  | 80(x) | 47(y) | 5 |
| Saudi Arabia | 2018 |  | 89 |  |  |  |  |  |  |  |  | 48 |  |  |  |  | 2 |
| *Total of indicators* | | *9* | *46* | *6* | *4* | *17* | *15* | *20* | *2* | *14* | *10* | *7* | *30* | *26* | *25* | *5* | *236* |
| *Median* | | *60* | *91* | *70* | *69* | *49* | *54* | *48* | *22* | *42* | *33* | *43* | *18* | *45* | *29* | *9* | *5* |
| *Min-Max* | | *32-92* | *60-98* | *16-87* | *37-85* | *21-89* | *13-77* | *12-72* | *10-34* | *11-66* | *8-62* | *8-65* | *0.1-57* | *24-78* | *0-80* | *2-47* | *1-12* |

* Number of indicators per country; †The highest prevalence of any breastfed available was used as a proxy of ever breastfed in specific cases; Breastfeeding rates extracted at: (‡) 2weeks; (a) 3 months; (b) 1 month (c) 2 months; (d) 6 weeks; (e) 6 weeks; (f) <6-weeks; (g) 0-2 months; (h) 6-8 weeks; (i)1-2 months; (m) 2-3 months; (n) 3-4 months; (p) 3-5 months; (q) 4-5 months; (r) <12 months; (s) 5-6 months; (t) 9-11 months; (v) >10-12 months; (x) 12-15 months; (z) 13-15 months; (y) 20-23 months; (w) 21-24.9 months.

**Table S2**. Annual growth rate of breastfeeding indicators of high-income countries and territories with comparable estimations across years.^1^

| **Country/indicator** | **First point in time** | | **Last point in time** | | **Annual change** |  | **Exclusive** | **First point in time** | | | | **Last point in time** | | | **Annual change** |
| --- | --- | --- | --- | --- | --- | --- | --- | --- | --- | --- | --- | --- | --- | --- | --- |
| **Ever breastfed** | **Year** | **%** | **Year** | **%** | **pts** |  | **at 6 months** | | **Year** | | **%** | **Year** | | **%** | **pts** |
| Puerto Rico | 2013 | 83 | 2015 | 86 | 1.5 |  | Puerto Rico | | 2013 | | 20 | 2015 | | 27 | 3.5 |
| Kuwait | 2011 | 81.8 | 2017 | 90 | 1.4 |  | Palau | | 2016 | | 46.7 | 2018 | | 52.4 | 2.9 |
| Wales | 2015 | 58 | 2018 | 61.8 | 1.3 |  | Virgin Islands | | 2014 | | 18 | 2015 | | 20 | 2.0 |
| Singapore | 2001 | 84.9 | 2011 | 96.1 | 1.1 |  | Wales | | 2015 | | 10.2 | 2018 | | 15.7 | 1.8 |
| Ireland. Rep | 2007 | 50.6 | 2016 | 59.9 | 1.0 |  | Uruguay | | 1996 | | 8.4 | 2011 | | 35.7 | 1.8 |
| Northern Ireland | 2007 | 50.6 | 2017 | 59.9 | 0.9 |  | Canada | | 2015 | | 32 | 2018 | | 37 | 1.7 |
| Scotland | 2016 | 63.3 | 2018 | 65.1 | 0.9 |  | Northern Ireland | | 2013 | | 6.9 | 2017 | | 13.5 | 1.7 |
| United States | 2000 | 70 | 2016 | 84 | 0.9 |  | Lithuania | | 2015 | | 32 | 2018 | | 36.7 | 1.6 |
| Korea, Rep | 2003 | 82 | 2018 | 94.2 | 0.8 |  | United States | | 2004 | | 12 | 2016 | | 25.4 | 1.1 |
| Canada | 2015 | 90 | 2018 | 91.9 | 0.6 |  | Aruba | | 2002 | | 0.1 | 2010 | | 9 | 1.1 |
| Malta | 2000 | 64 | 2018 | 71 | 0.4 |  | Spain | | 1995 | | 15.1 | 2017 | | 39 | 1.1 |
| Antigua and Barbuda | 2000 | 86 | 2006 | 88 | 0.3 |  | Chile | | 2005 | | 46 | 2016 | | 57 | 1.0 |
| Australia | 1995 | 86 | 2018 | 92.7 | 0.3 |  | Guam | | 2014 | | 24 | 2016 | | 26 | 1.0 |
| New Zealand | 2008 | 90.9 | 2017 | 93.1 | 0.2 |  | Iceland | | 2004 | | 5 | 2008 | | 9 | 1.0 |
| Cyprus | 2014 | 90.9 | 2017 | 91.2 | 0.1 |  | Switzerland | | 2003 | | 21 | 2014 | | 26 | 0.5 |
| Switzerland | 2003 | 94 | 2014 | 95 | 0.1 |  | Finland | | 1995 | | 0 | 2019 | | 9 | 0.4 |
| Japan | 1985 | 90.9 | 2015 | 93.5 | 0.1 |  | Singapore | | 2001 | | 0.3 | 2011 | | 0.8 | 0.1 |
| England | 2011 | 73.7 | 2018 | 74 | 0.0 |  | Greece | | 2007 | | 1 | 2017 | | 1 | 0.0 |
| Latvia | 2008 | 91 | 2019 | 91.3 | 0.0 |  | Australia | | 2015 | | 29 | 2018 | | 28.9 | 0.0 |
| Luxembourg | 2008 | 90 | 2015 | 90 | 0.0 |  | Czech Republic | | 2000 | | 44 | 2010 | | 43 | -0.1 |
| Czech Republic | 2000 | 95.1 | 2017 | 94.8 | 0.0 |  | Luxembourg | | 2008 | | 5 | 2015 | | 3 | -0.3 |
| Uruguay | 2007 | 98.5 | 2011 | 98.4 | 0.0 |  | Sweden | | 1986 | | 25.6 | 2017 | | 13 | -0.4 |
| Norway | 2006 | 98 | 2019 | 97 | -0.1 |  | Norway | | 2006 | | 9 | 2019 | | 2 | -0.5 |
| Sweden | 1986 | 97.2 | 2017 | 94.6 | -0.1 |  | Korea, Rep | | 2003 | | 25.3 | 2018 | | 2 | -1.6 |
| Bahrain | 1995 | 96 | 2002 | 95 | -0.1 |  | Estonia | | 2013 | | 41.7 | 2019 | | 23.1 | -3.1 |
| Croatia | 2005 | 97.3 | 2016 | 94.9 | -0.2 |  |  | |  | |  |  | |  |  |
| Iceland | 2004 | 99 | 2008 | 98 | -0.3 |  |  | |  | |  |  | |  |  |
| France | 2010 | 69 | 2016 | 67 | -0.3 |  |  | |  | |  |  | |  |  |
| *continued* |  |  |  |  |  |  |  | |  | |  |  | |  |  |
|  |  |  |  |  |  |  |  | |  | |  |  | |  |  |
|  |  |  |  |  |  |  |  | |  | |  |  | |  |  |
| ***continued Ever breastfed*** | **Year** | **%** | **Year** | **%** | **pts** |  | **Any breastfeeding** | | **First point in time** | | | **Last point in time** | | | **Annual change** |
| Oman | 2009 | 99.6 | 2017 | 96.1 | -0.4 |  | **at 6 months** | | **Year** | | **%** | **Year** | | **%** | **pts** |
| Denmark | 2012 | 89.4 | 2017 | 86.8 | -0.5 |  | Puerto Rico | | 2013 | | 37 | 2015 | | 47 | 5.0 |
| Aruba | 2002 | 83 | 2010 | 75 | -1.0 |  | Estonia | | 2013 | | 20.7 | 2019 | | 48.7 | 4.7 |
| Palau | 2016 | 100 | 2018 | 97.7 | -1.2 |  | Greece | | 2007 | | 21 | 2017 | | 45 | 2.4 |
| Estonia | 2013 | 93.4 | 2019 | 84.9 | -1.4 |  | Singapore | | 2001 | | 21.1 | 2011 | | 41.6 | 2.1 |
| Guam | 2014 | 86 | 2016 | 83 | -1.5 |  | Aruba | | 2002 | | 20 | 2010 | | 35 | 1.9 |
| Virgin Islands | 2014 | 86 | 2015 | 84 | -2.0 |  | Wales | | 2015 | | 17.5 | 2018 | | 22.7 | 1.7 |
| **Continued Breastfeeding** | **First point in time** | | **Last point in time** | | **Annual change** |  | Finland | | 1995 | | 40 | 2019 | | 76 | 1.5 |
| **at 12 months** | **Year** | **%** | **Year** | **%** | pts |  | United States | | 2000 | | 35 | 2016 | | 57 | 1.4 |
| Virgin Islands | 2014 | 28 | 2015 | 33 | 5.0 |  | Luxembourg | | 2008 | | 36 | 2015 | | 45 | 1.3 |
| Puerto Rico | 2013 | 21 | 2015 | 30 | 4.5 |  | Guam | | 2014 | | 56 | 2016 | | 58 | 1.0 |
| Guam | 2014 | 31 | 2016 | 40 | 4.5 |  | Spain | | 1995 | | 33.2 | 2011 | | 47 | 0.9 |
| Luxembourg | 2008 | 12 | 2015 | 24 | 1.7 |  | Czech Republic | | 2000 | | 23.8 | 2017 | | 38 | 0.8 |
| Finland | 1995 | 25 | 2019 | 62 | 1.5 |  | Iceland | | 2004 | | 72 | 2008 | | 75 | 0.8 |
| Iceland | 2004 | 23.3 | 2008 | 29 | 1.5 |  | Sweden | | 1986 | | 50.7 | 2017 | | 63 | 0.4 |
| Estonia | 2013 | 25 | 2019 | 33 | 1.4 |  | Portugal | | 2006 | | 28.6 | 2014 | | 30.3 | 0.2 |
| Uruguay | 1996 | 24.6 | 2011 | 45 | 1.3 |  | Korea, Rep | | 2006 | | 25.2 | 2018 | | 26 | 0.1 |
| Northern Ireland | 2013 | 5.2 | 2017 | 11 | 1.3 |  | Switzerland | | 2003 | | 27 | 2014 | | 27 | 0.0 |
| United States | 2000 | 16 | 2016 | 36.2 | 1.3 |  | Northern Ireland | | 2013 | | 3.9 | 2017 | | 3.6 | -0.1 |
| Latvia | 2008 | 19 | 2019 | 27 | 0.7 |  | Norway | | 2006 | | 80 | 2019 | | 78 | -0.2 |
| Norway | 1999 | 36 | 2019 | 48 | 0.6 |  | Australia | | 2015 | | 35.8 | 2018 | | 34.1 | -0.6 |
| Sweden | 2002 | 19.9 | 2017 | 27 | 0.5 |  | Virgin Islands | | 2014 | | 53 | 2015 | | 52 | -1.0 |
| Bahrain | 1995 | 64 | 2002 | 65.3 | 0.2 |  |  | |  | |  |  | |  |  |
| Oman | 2009 | 78.9 | 2017 | 80 | 0.1 |  |  | |  | |  |  | |  |  |
| Switzerland | 2003 | 0 | 2014 | 0 | 0.0 |  |  | |  | |  |  | |  |  |
| Korea, Rep | 2006 | 23.7 | 2018 | 7.3 | -1.4 |  | **Early breastfeeding** | | **First point in time** | | | **Last point in time** | | | **Annual change** |
| Scotland | 2017 | 19.9 | 2018 | 18 | -1.9 |  | **(1h)** | | **Year** | **%** | | **Year** | **%** | | pts |
| **Continued breastfeeding** | **First point in time** | | **Last point in time** | | **Annual change** |  | Korea, Rep | | 2015 | | 18.1 | 2018 | | 31.8 | 4.6 |
| **At 24 months** | **Year** | **%** | **Year** | **Year** | **pts** |  | Malta | | 2000 | | 41 | 2003 | | 49 | 2.7 |
| Uruguay | 1996 | 11.8 | 2011 | 27 | 1.0 |  | Luxembourg | | 2008 | | 66.5 | 2015 | | 71.2 | 0.7 |
| Oman | 2009 | 44 | 2017 | 47 | 0.4 |  | Oman | | 2009 | | 82.6 | 2017 | | 82 | -0.1 |
| Norway | 1999 | 4 | 2019 | 8 | 0.2 |  | Uruguay | | 2007 | | 60.1 | 2011 | | 59.3 | -0.2 |
|  |  |  |  |  |  |  | Latvia | | 2014 | | 81 | 2015 | | 80 | -1.0 |

^1^*Note:* Annual growth calculated by the difference between the last and first annual rate divided by the difference in years between rates. Analyses were restricted to countries without differences in methodology that limited comparisons between years.

***continued***

| **Exclusive** | **First point in time** | | **Last point in time** | | **Annual change** |
| --- | --- | --- | --- | --- | --- |
| **at 3 months** | **Year** | **%** | **Year** | **%** | **pts** |
| Puerto Rico | 2013 | 35 | 2015 | 48 | 6.5 |
| Guam | 2014 | 41 | 2016 | 47 | 3.0 |
| Uruguay | 1996 | 31.4 | 2011 | 72 | 2.7 |
| Iceland | 2004 | 61.7 | 2008 | 69 | 1.8 |
| Lithuania | 2015 | 49 | 2018 | 53 | 1.3 |
| United States | 2004 | 32 | 2016 | 48 | 1.3 |
| Spain | 1995 | 37 | 2017 | 64 | 1.2 |
| Finland | 1995 | 26 | 2019 | 53 | 1.1 |
| Northern Ireland | 2013 | 14.5 | 2017 | 19 | 1.1 |
| Aruba | 2002 | 7 | 2010 | 12 | 0.6 |
| Portugal | 2006 | 51.9 | 2014 | 55.9 | 0.5 |
| Norway | 2006 | 63 | 2019 | 68 | 0.4 |
| Korea, Republic | 2003 | 34.1 | 2018 | 31 | -0.2 |
| Estonia | 2013 | 67.2 | 2019 | 65.3 | -0.3 |
| Czech Republic | 2000 | 54 | 2017 | 21 | -1.9 |
| Virgin Island | 2014 | 35 | 2015 | 32 | -3.0 |

| **Exclusive** | **First point in time** | | **Last point in time** | | **Annual change** |
| --- | --- | --- | --- | --- | --- |
| **at 4 months** | **Year** | **%** | **Year** | **%** | **pts** |
| Uruguay | 1996 | 22 | 2011 | 66 | 2.9 |
| Finland | 1995 | 10 | 2019 | 50 | 1.7 |
| Portugal | 2006 | 41.2 | 2014 | 48.5 | 0.9 |
| Aruba | 2002 | 4 | 2010 | 11 | 0.9 |
| Sweden | 1986 | 30.3 | 2017 | 49.8 | 0.6 |
| Australia | 2015 | 57.5 | 2018 | 58 | 0.2 |
| Korea, Rep | 2003 | 30.6 | 2018 | 26 | -0.3 |
| Norway | 2006 | 46 | 2019 | 39 | -0.5 |

*^1^ Note:* Annual growth calculated by the difference between the last and first annual rate divided by the difference in years between rates.

Analyses were restricted to countries without differences in methodology that limited comparisons between years.
